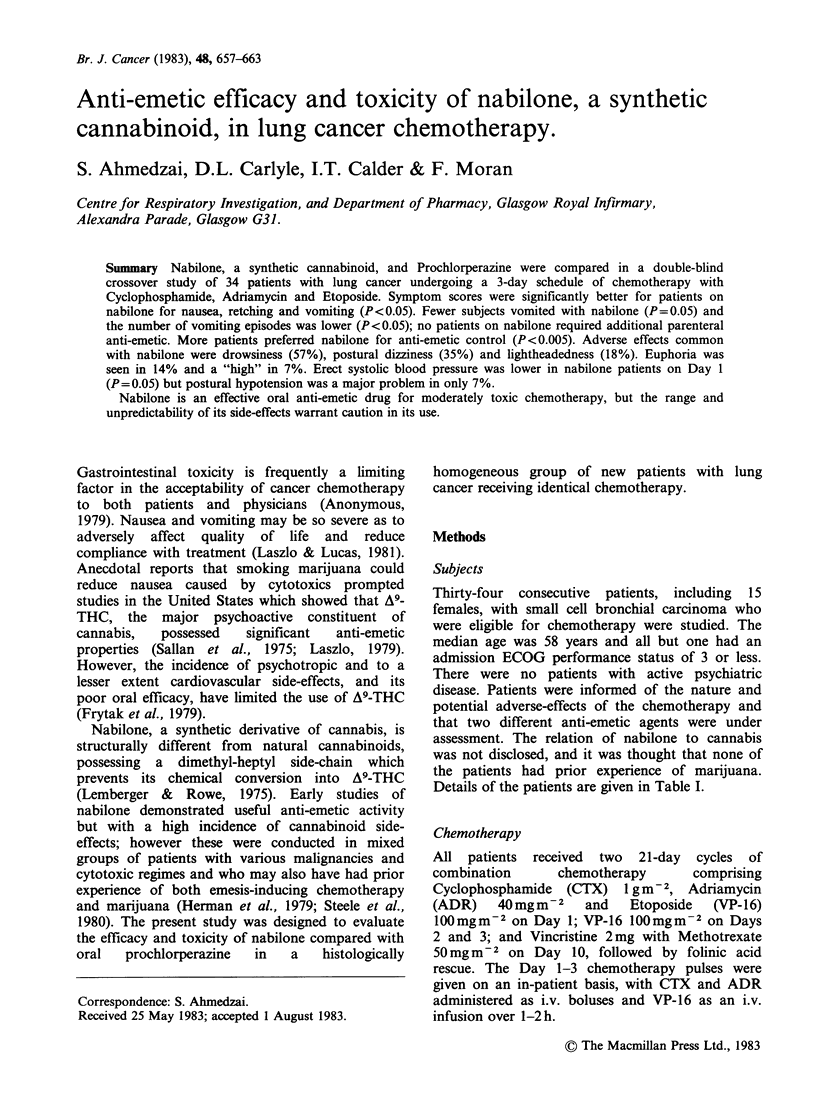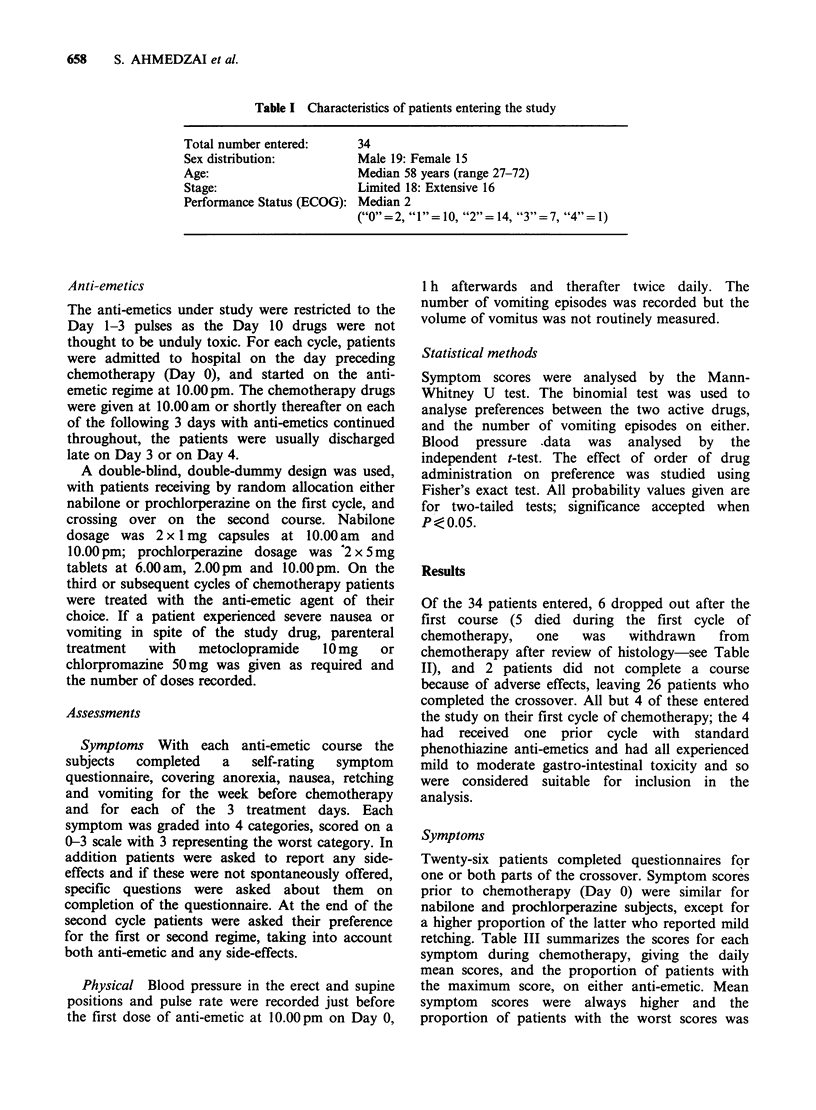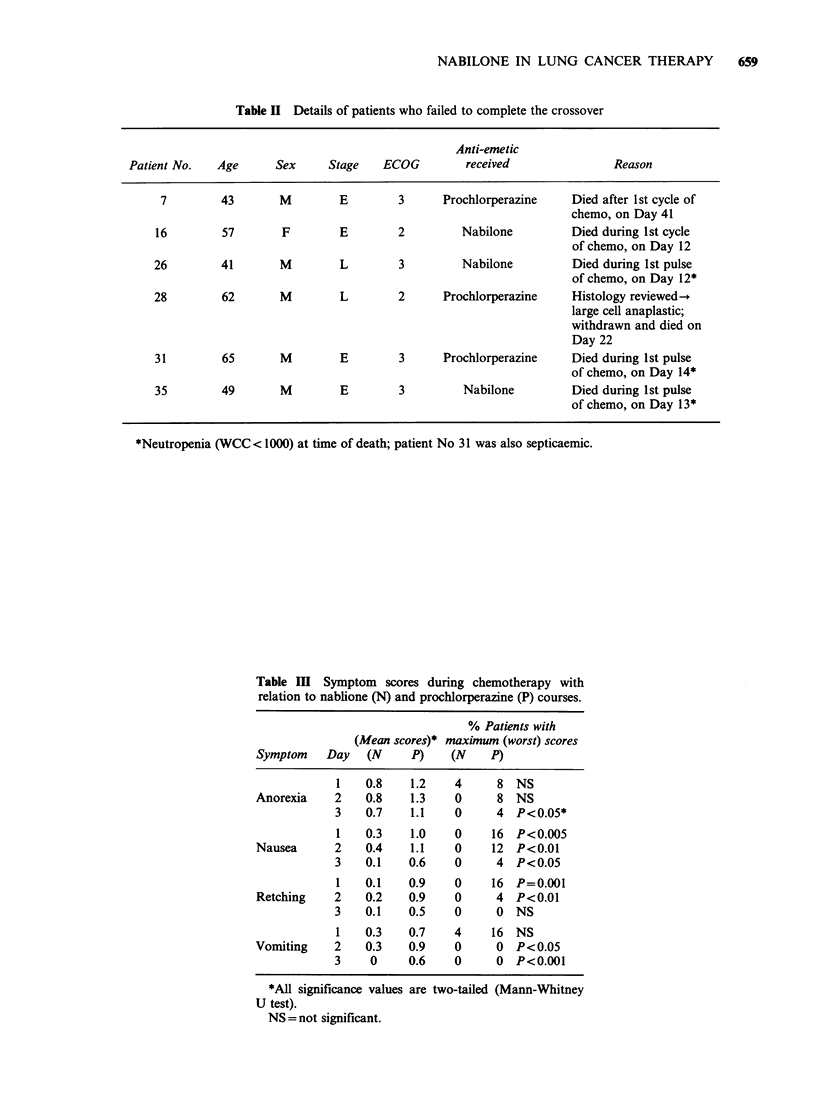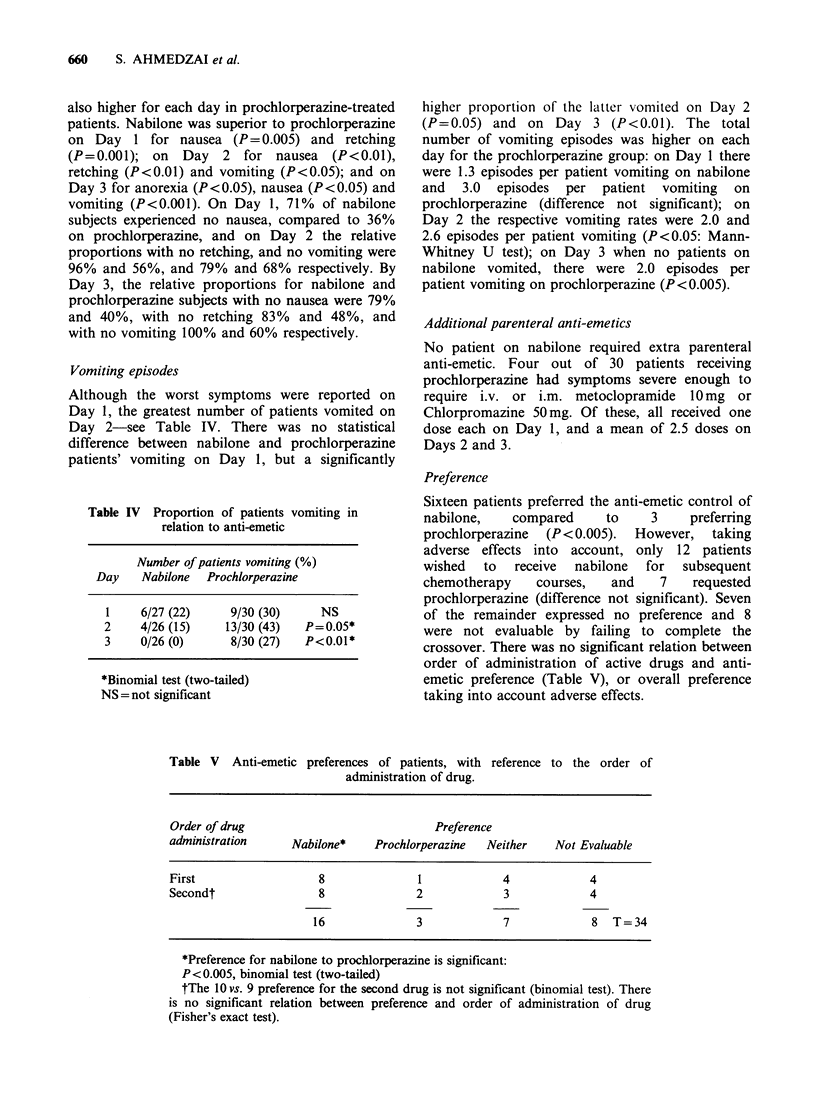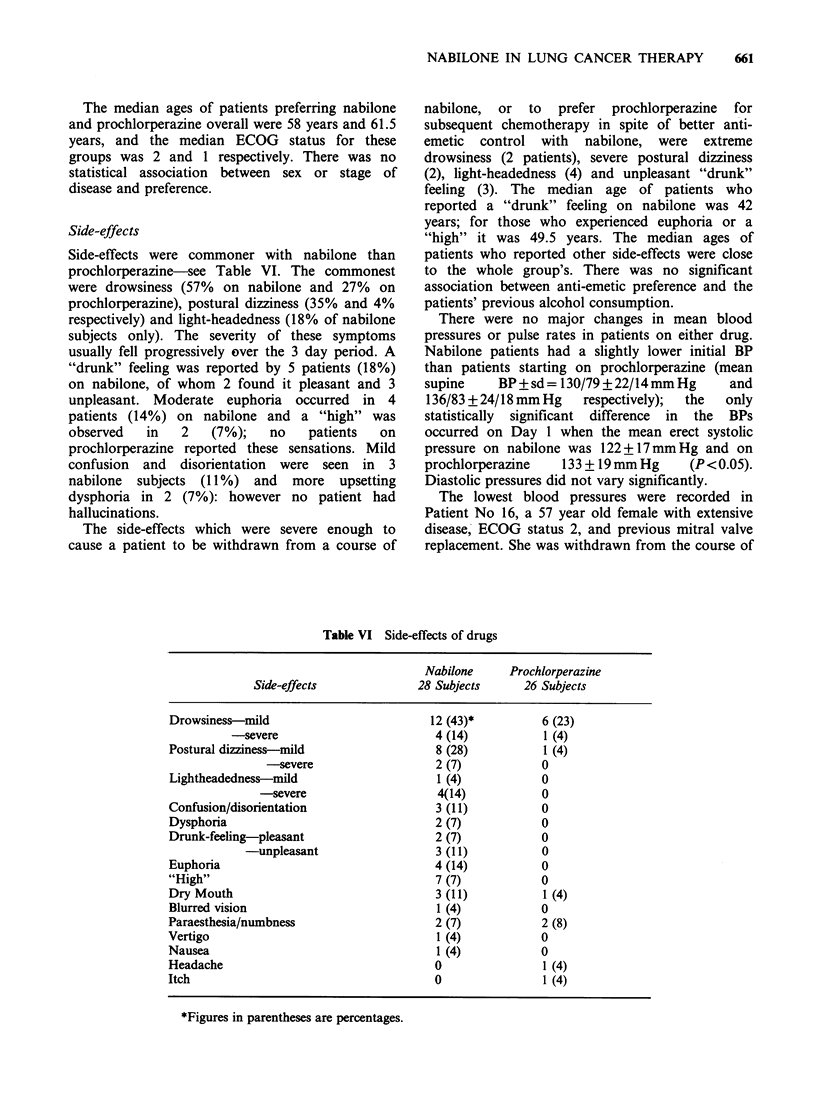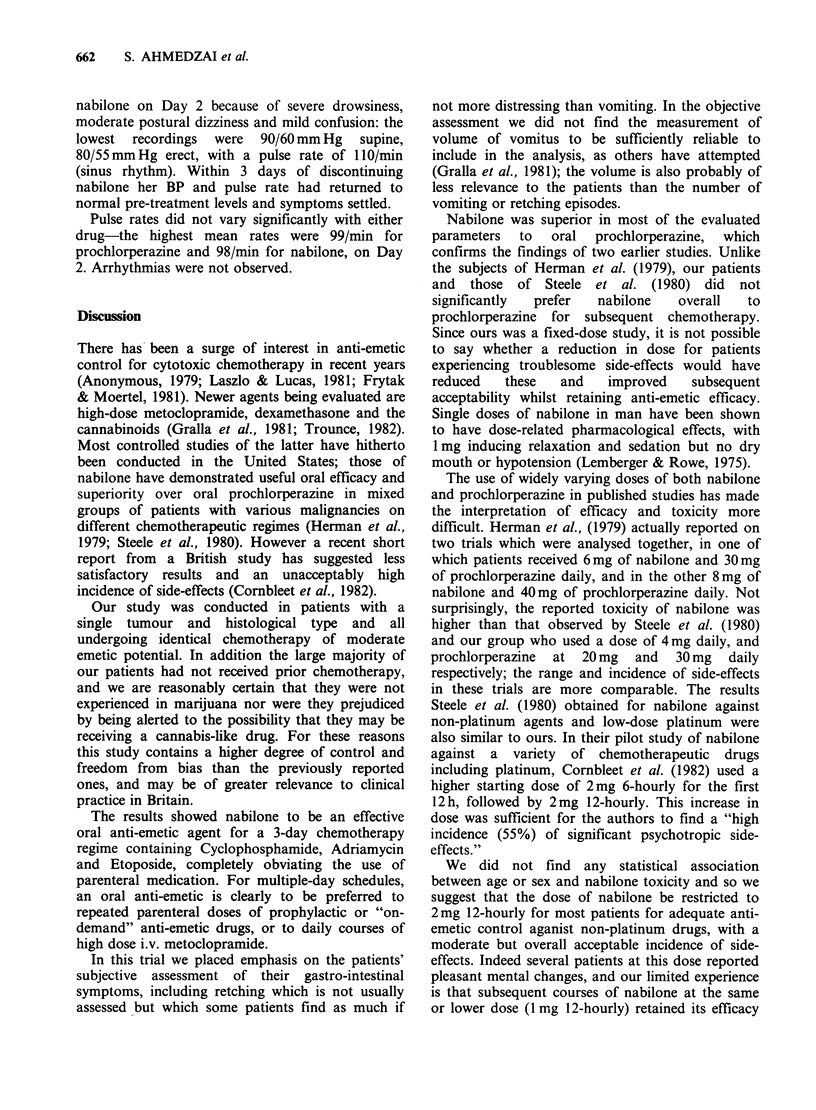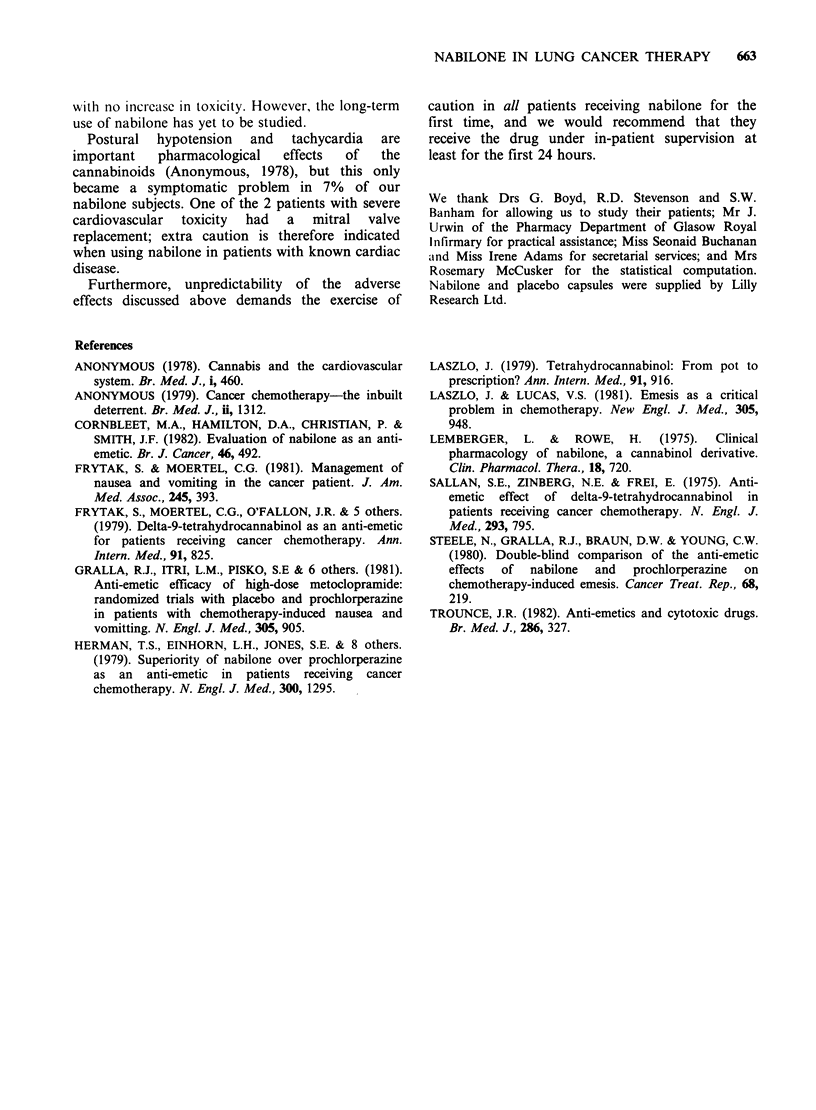# Anti-emetic efficacy and toxicity of nabilone, a synthetic cannabinoid, in lung cancer chemotherapy.

**DOI:** 10.1038/bjc.1983.247

**Published:** 1983-11

**Authors:** S. Ahmedzai, D. L. Carlyle, I. T. Calder, F. Moran

## Abstract

Nabilone, a synthetic cannabinoid, and Prochlorperazine were compared in a double-blind crossover study of 34 patients with lung cancer undergoing a 3-day schedule of chemotherapy with Cyclophosphamide, Adriamycin and Etoposide. Symptom scores were significantly better for patients on nabilone for nausea, retching and vomiting (P less than 0.05). Fewer subjects vomited with nabilone (P = 0.05) and the number of vomiting episodes was lower (P less than 0.05); no patients on nabilone required additional parenteral anti-emetic. More patients preferred nabilone for anti-emetic control (P less than 0.005). Adverse effects common with nabilone were drowsiness (57%), postural dizziness (35%) and lightheadedness (18%). Euphoria was seen in 14% and a "high" in 7%. Erect systolic blood pressure was lower in nabilone patients on Day 1 (P = 0.05) but postural hypotension was a major problem in only 7%. Nabilone is an effective oral anti-emetic drug for moderately toxic chemotherapy, but the range and unpredictability of its side-effects warrant caution in its use.


Br. J. Cancer (1983), 48, 657–663

### Anti-emetic efficacy and toxicity of nabilone, a synthetic cannabinoid, in lung cancer chemotherapy.

S. Ahmedzai, D.L. Carlyle, I.T. Calder & F. Moran

*Centre for Respiratory Investigation, and Department of Pharmacy, Glasgow Royal Infirmary, Alexandra Parade, Glasgow G31.*

**Summary** Nabilone, a synthetic cannabinoid, and Prochlorperazine were compared in a double-blind crossover study of 34 patients with lung cancer undergoing a 3-day schedule of chemotherapy with Cyclophosphamide, Adriamycin and Etoposide. Symptom scores were significantly better for patients on nabilone for nausea, retching and vomiting ($P<0.05$). Fewer subjects vomited with nabilone ($P=0.05$) and the number of vomiting episodes was lower ($P<0.05$); no patients on nabilone required additional parenteral anti-emetic. More patients preferred nabilone for anti-emetic control ($P<0.005$). Adverse effects common with nabilone were drowsiness (57%), postural dizziness (35%) and lightheadedness (18%). Euphoria was seen in 14% and a "high" in 7%. Erect systolic blood pressure was lower in nabilone patients on Day 1 ($P=0.05$) but postural hypotension was a major problem in only 7%.

Nabilone is an effective oral anti-emetic drug for moderately toxic chemotherapy, but the range and unpredictability of its side-effects warrant caution in its use.

Gastrointestinal toxicity is frequently a limiting factor in the acceptability of cancer chemotherapy to both patients and physicians (Anonymous, 1979). Nausea and vomiting may be so severe as to adversely affect quality of life and reduce compliance with treatment (Laszlo & Lucas, 1981). Anecdotal reports that smoking marijuana could reduce nausea caused by cytotoxics prompted studies in the United States which showed that $\Delta^9$-THC, the major psychoactive constituent of cannabis, possessed significant anti-emetic properties (Sallan *et al.*, 1975; Laszlo, 1979). However, the incidence of psychotropic and to a lesser extent cardiovascular side-effects, and its poor oral efficacy, have limited the use of $\Delta^9$-THC (Frytak *et al.*, 1979).

Nabilone, a synthetic derivative of cannabis, is structurally different from natural cannabinoids, possessing a dimethyl-heptyl side-chain which prevents its chemical conversion into $\Delta^9$-THC (Lemberger & Rowe, 1975). Early studies of nabilone demonstrated useful anti-emetic activity but with a high incidence of cannabinoid side-effects; however these were conducted in mixed groups of patients with various malignancies and cytotoxic regimes and who may also have had prior experience of both emesis-inducing chemotherapy and marijuana (Herman *et al.*, 1979; Steele *et al.*, 1980). The present study was designed to evaluate the efficacy and toxicity of nabilone compared with oral prochlorperazine in a histologically homogeneous group of new patients with lung cancer receiving identical chemotherapy.

Correspondence: S. Ahmedzai.
Received 25 May 1983; accepted 1 August 1983.

### Methods

### *Subjects*

Thirty-four consecutive patients, including 15 females, with small cell bronchial carcinoma who were eligible for chemotherapy were studied. The median age was 58 years and all but one had an admission ECOG performance status of 3 or less. There were no patients with active psychiatric disease. Patients were informed of the nature and potential adverse-effects of the chemotherapy and that two different anti-emetic agents were under assessment. The relation of nabilone to cannabis was not disclosed, and it was thought that none of the patients had prior experience of marijuana. Details of the patients are given in Table I.

### *Chemotherapy*

All patients received two 21-day cycles of combination chemotherapy comprising Cyclophosphamide (CTX) $1\,g\,m^{-2}$, Adriamycin (ADR) $40\,mg\,m^{-2}$ and Etoposide (VP-16) $100\,mg\,m^{-2}$ on Day 1; VP-16 $100\,mg\,m^{-2}$ on Days 2 and 3; and Vincristine 2 mg with Methotrexate $50\,mg\,m^{-2}$ on Day 10, followed by folinic acid rescue. The Day 1–3 chemotherapy pulses were given on an in-patient basis, with CTX and ADR administered as i.v. boluses and VP-16 as an i.v. infusion over 1–2 h.

© The Macmillan Press Ltd., 1983

**Table I** Characteristics of patients entering the study

| | |
|---|---|
| Total number entered: | 34 |
| Sex distribution: | Male 19: Female 15 |
| Age: | Median 58 years (range 27–72) |
| Stage: | Limited 18: Extensive 16 |
| Performance Status (ECOG): | Median 2<br>("0" = 2, "1" = 10, "2" = 14, "3" = 7, "4" = 1) |

*Anti-emetics*

The anti-emetics under study were restricted to the Day 1–3 pulses as the Day 10 drugs were not thought to be unduly toxic. For each cycle, patients were admitted to hospital on the day preceding chemotherapy (Day 0), and started on the anti-emetic regime at 10.00 pm. The chemotherapy drugs were given at 10.00 am or shortly thereafter on each of the following 3 days with anti-emetics continued throughout, the patients were usually discharged late on Day 3 or on Day 4.

A double-blind, double-dummy design was used, with patients receiving by random allocation either nabilone or prochlorperazine on the first cycle, and crossing over on the second course. Nabilone dosage was 2 × 1 mg capsules at 10.00 am and 10.00 pm; prochlorperazine dosage was 2 × 5 mg tablets at 6.00 am, 2.00 pm and 10.00 pm. On the third or subsequent cycles of chemotherapy patients were treated with the anti-emetic agent of their choice. If a patient experienced severe nausea or vomiting in spite of the study drug, parenteral treatment with metoclopramide 10 mg or chlorpromazine 50 mg was given as required and the number of doses recorded.

*Assessments*

*Symptoms* With each anti-emetic course the subjects completed a self-rating symptom questionnaire, covering anorexia, nausea, retching and vomiting for the week before chemotherapy and for each of the 3 treatment days. Each symptom was graded into 4 categories, scored on a 0–3 scale with 3 representing the worst category. In addition patients were asked to report any side-effects and if these were not spontaneously offered, specific questions were asked about them on completion of the questionnaire. At the end of the second cycle patients were asked their preference for the first or second regime, taking into account both anti-emetic and any side-effects.

*Physical* Blood pressure in the erect and supine positions and pulse rate were recorded just before the first dose of anti-emetic at 10.00 pm on Day 0, 1 h afterwards and therafter twice daily. The number of vomiting episodes was recorded but the volume of vomitus was not routinely measured.

*Statistical methods*

Symptom scores were analysed by the Mann-Whitney U test. The binomial test was used to analyse preferences between the two active drugs, and the number of vomiting episodes on either. Blood pressure data was analysed by the independent *t*-test. The effect of order of drug administration on preference was studied using Fisher's exact test. All probability values given are for two-tailed tests; significance accepted when $P \leqslant 0.05$.

### Results

Of the 34 patients entered, 6 dropped out after the first course (5 died during the first cycle of chemotherapy, one was withdrawn from chemotherapy after review of histology—see Table II), and 2 patients did not complete a course because of adverse effects, leaving 26 patients who completed the crossover. All but 4 of these entered the study on their first cycle of chemotherapy; the 4 had received one prior cycle with standard phenothiazine anti-emetics and had all experienced mild to moderate gastro-intestinal toxicity and so were considered suitable for inclusion in the analysis.

*Symptoms*

Twenty-six patients completed questionnaires for one or both parts of the crossover. Symptom scores prior to chemotherapy (Day 0) were similar for nabilone and prochlorperazine subjects, except for a higher proportion of the latter who reported mild retching. Table III summarizes the scores for each symptom during chemotherapy, giving the daily mean scores, and the proportion of patients with the maximum score, on either anti-emetic. Mean symptom scores were always higher and the proportion of patients with the worst scores was

**Table II** Details of patients who failed to complete the crossover

| *Patient No.* | *Age* | *Sex* | *Stage* | *ECOG* | *Anti-emetic received* | *Reason* |
|---|---|---|---|---|---|---|
| 7 | 43 | M | E | 3 | Prochlorperazine | Died after 1st cycle of chemo, on Day 41 |
| 16 | 57 | F | E | 2 | Nabilone | Died during 1st cycle of chemo, on Day 12 |
| 26 | 41 | M | L | 3 | Nabilone | Died during 1st pulse of chemo, on Day 12* |
| 28 | 62 | M | L | 2 | Prochlorperazine | Histology reviewed→ large cell anaplastic; withdrawn and died on Day 22 |
| 31 | 65 | M | E | 3 | Prochlorperazine | Died during 1st pulse of chemo, on Day 14* |
| 35 | 49 | M | E | 3 | Nabilone | Died during 1st pulse of chemo, on Day 13* |

*Neutropenia (WCC < 1000) at time of death; patient No 31 was also septicaemic.

**Table III** Symptom scores during chemotherapy with relation to nablione (N) and prochlorperazine (P) courses.

| *Symptom* | *Day* | *(Mean scores)** *(N* | *P)* | *% Patients with maximum (worst) scores* *(N* | *P)* | |
|---|---|---|---|---|---|---|
| | 1 | 0.8 | 1.2 | 4 | 8 | NS |
| Anorexia | 2 | 0.8 | 1.3 | 0 | 8 | NS |
| | 3 | 0.7 | 1.1 | 0 | 4 | $P<0.05$* |
| | 1 | 0.3 | 1.0 | 0 | 16 | $P<0.005$ |
| Nausea | 2 | 0.4 | 1.1 | 0 | 12 | $P<0.01$ |
| | 3 | 0.1 | 0.6 | 0 | 4 | $P<0.05$ |
| | 1 | 0.1 | 0.9 | 0 | 16 | $P=0.001$ |
| Retching | 2 | 0.2 | 0.9 | 0 | 4 | $P<0.01$ |
| | 3 | 0.1 | 0.5 | 0 | 0 | NS |
| | 1 | 0.3 | 0.7 | 4 | 16 | NS |
| Vomiting | 2 | 0.3 | 0.9 | 0 | 0 | $P<0.05$ |
| | 3 | 0 | 0.6 | 0 | 0 | $P<0.001$ |

*All significance values are two-tailed (Mann-Whitney U test).

NS = not significant.

also higher for each day in prochlorperazine-treated patients. Nabilone was superior to prochlorperazine on Day 1 for nausea ($P=0.005$) and retching ($P=0.001$); on Day 2 for nausea ($P<0.01$), retching ($P<0.01$) and vomiting ($P<0.05$); and on Day 3 for anorexia ($P<0.05$), nausea ($P<0.05$) and vomiting ($P<0.001$). On Day 1, 71% of nabilone subjects experienced no nausea, compared to 36% on prochlorperazine, and on Day 2 the relative proportions with no retching, and no vomiting were 96% and 56%, and 79% and 68% respectively. By Day 3, the relative proportions for nabilone and prochlorperazine subjects with no nausea were 79% and 40%, with no retching 83% and 48%, and with no vomiting 100% and 60% respectively.

*Vomiting episodes*

Although the worst symptoms were reported on Day 1, the greatest number of patients vomited on Day 2—see Table IV. There was no statistical difference between nabilone and prochlorperazine patients' vomiting on Day 1, but a significantly higher proportion of the latter vomited on Day 2 ($P=0.05$) and on Day 3 ($P<0.01$). The total number of vomiting episodes was higher on each day for the prochlorperazine group: on Day 1 there were 1.3 episodes per patient vomiting on nabilone and 3.0 episodes per patient vomiting on prochlorperazine (difference not significant); on Day 2 the respective vomiting rates were 2.0 and 2.6 episodes per patient vomiting ($P<0.05$: Mann-Whitney U test); on Day 3 when no patients on nabilone vomited, there were 2.0 episodes per patient vomiting on prochlorperazine ($P<0.005$).

**Table IV** Proportion of patients vomiting in relation to anti-emetic

| | *Number of patients vomiting* (%) | | |
|---|---|---|---|
| *Day* | *Nabilone* | *Prochlorperazine* | |
| 1 | 6/27 (22) | 9/30 (30) | NS |
| 2 | 4/26 (15) | 13/30 (43) | $P=0.05$* |
| 3 | 0/26 (0) | 8/30 (27) | $P<0.01$* |

*Binomial test (two-tailed)
NS = not significant

*Additional parenteral anti-emetics*

No patient on nabilone required extra parenteral anti-emetic. Four out of 30 patients receiving prochlorperazine had symptoms severe enough to require i.v. or i.m. metoclopramide 10 mg or Chlorpromazine 50 mg. Of these, all received one dose each on Day 1, and a mean of 2.5 doses on Days 2 and 3.

*Preference*

Sixteen patients preferred the anti-emetic control of nabilone, compared to 3 preferring prochlorperazine ($P<0.005$). However, taking adverse effects into account, only 12 patients wished to receive nabilone for subsequent chemotherapy courses, and 7 requested prochlorperazine (difference not significant). Seven of the remainder expressed no preference and 8 were not evaluable by failing to complete the crossover. There was no significant relation between order of administration of active drugs and anti-emetic preference (Table V), or overall preference taking into account adverse effects.

**Table V** Anti-emetic preferences of patients, with reference to the order of administration of drug.

| *Order of drug administration* | *Preference* | | | | |
|---|---|---|---|---|---|
| | *Nabilone** | *Prochlorperazine* | *Neither* | *Not Evaluable* | |
| First | 8 | 1 | 4 | 4 | |
| Second† | 8 | 2 | 3 | 4 | |
| | 16 | 3 | 7 | 8 | T=34 |

*Preference for nabilone to prochlorperazine is significant:
$P<0.005$, binomial test (two-tailed)

†The 10 *vs.* 9 preference for the second drug is not significant (binomial test). There is no significant relation between preference and order of administration of drug (Fisher's exact test).

The median ages of patients preferring nabilone and prochlorperazine overall were 58 years and 61.5 years, and the median ECOG status for these groups was 2 and 1 respectively. There was no statistical association between sex or stage of disease and preference.

*Side-effects*

Side-effects were commoner with nabilone than prochlorperazine—see Table VI. The commonest were drowsiness (57% on nabilone and 27% on prochlorperazine), postural dizziness (35% and 4% respectively) and light-headedness (18% of nabilone subjects only). The severity of these symptoms usually fell progressively over the 3 day period. A "drunk" feeling was reported by 5 patients (18%) on nabilone, of whom 2 found it pleasant and 3 unpleasant. Moderate euphoria occurred in 4 patients (14%) on nabilone and a "high" was observed in 2 (7%); no patients on prochlorperazine reported these sensations. Mild confusion and disorientation were seen in 3 nabilone subjects (11%) and more upsetting dysphoria in 2 (7%): however no patient had hallucinations.

The side-effects which were severe enough to cause a patient to be withdrawn from a course of nabilone, or to prefer prochlorperazine for subsequent chemotherapy in spite of better anti-emetic control with nabilone, were extreme drowsiness (2 patients), severe postural dizziness (2), light-headedness (4) and unpleasant "drunk" feeling (3). The median age of patients who reported a "drunk" feeling on nabilone was 42 years; for those who experienced euphoria or a "high" it was 49.5 years. The median ages of patients who reported other side-effects were close to the whole group's. There was no significant association between anti-emetic preference and the patients' previous alcohol consumption.

There were no major changes in mean blood pressures or pulse rates in patients on either drug. Nabilone patients had a slightly lower initial BP than patients starting on prochlorperazine (mean supine BP $\pm$ sd = 130/79 $\pm$ 22/14 mm Hg and 136/83 $\pm$ 24/18 mm Hg respectively); the only statistically significant difference in the BPs occurred on Day 1 when the mean erect systolic pressure on nabilone was 122 $\pm$ 17 mm Hg and on prochlorperazine 133 $\pm$ 19 mm Hg ($P < 0.05$). Diastolic pressures did not vary significantly.

The lowest blood pressures were recorded in Patient No 16, a 57 year old female with extensive disease, ECOG status 2, and previous mitral valve replacement. She was withdrawn from the course of

**Table VI** Side-effects of drugs

| *Side-effects* | *Nabilone* 28 *Subjects* | *Prochlorperazine* 26 *Subjects* |
|---|---|---|
| Drowsiness—mild | 12 (43)* | 6 (23) |
| —severe | 4 (14) | 1 (4) |
| Postural dizziness—mild | 8 (28) | 1 (4) |
| —severe | 2 (7) | 0 |
| Lightheadedness—mild | 1 (4) | 0 |
| —severe | 4(14) | 0 |
| Confusion/disorientation | 3 (11) | 0 |
| Dysphoria | 2 (7) | 0 |
| Drunk-feeling—pleasant | 2 (7) | 0 |
| —unpleasant | 3 (11) | 0 |
| Euphoria | 4 (14) | 0 |
| "High" | 7 (7) | 0 |
| Dry Mouth | 3 (11) | 1 (4) |
| Blurred vision | 1 (4) | 0 |
| Paraesthesia/numbness | 2 (7) | 2 (8) |
| Vertigo | 1 (4) | 0 |
| Nausea | 1 (4) | 0 |
| Headache | 0 | 1 (4) |
| Itch | 0 | 1 (4) |

*Figures in parentheses are percentages.

nabilone on Day 2 because of severe drowsiness, moderate postural dizziness and mild confusion: the lowest recordings were 90/60 mm Hg supine, 80/55 mm Hg erect, with a pulse rate of 110/min (sinus rhythm). Within 3 days of discontinuing nabilone her BP and pulse rate had returned to normal pre-treatment levels and symptoms settled.

Pulse rates did not vary significantly with either drug—the highest mean rates were 99/min for prochlorperazine and 98/min for nabilone, on Day 2. Arrhythmias were not observed.

### Discussion

There has been a surge of interest in anti-emetic control for cytotoxic chemotherapy in recent years (Anonymous, 1979; Laszlo & Lucas, 1981; Frytak & Moertel, 1981). Newer agents being evaluated are high-dose metoclopramide, dexamethasone and the cannabinoids (Gralla *et al.*, 1981; Trounce, 1982). Most controlled studies of the latter have hitherto been conducted in the United States; those of nabilone have demonstrated useful oral efficacy and superiority over oral prochlorperazine in mixed groups of patients with various malignancies on different chemotherapeutic regimes (Herman *et al.*, 1979; Steele *et al.*, 1980). However a recent short report from a British study has suggested less satisfactory results and an unacceptably high incidence of side-effects (Cornbleet *et al.*, 1982).

Our study was conducted in patients with a single tumour and histological type and all undergoing identical chemotherapy of moderate emetic potential. In addition the large majority of our patients had not received prior chemotherapy, and we are reasonably certain that they were not experienced in marijuana nor were they prejudiced by being alerted to the possibility that they may be receiving a cannabis-like drug. For these reasons this study contains a higher degree of control and freedom from bias than the previously reported ones, and may be of greater relevance to clinical practice in Britain.

The results showed nabilone to be an effective oral anti-emetic agent for a 3-day chemotherapy regime containing Cyclophosphamide, Adriamycin and Etoposide, completely obviating the use of parenteral medication. For multiple-day schedules, an oral anti-emetic is clearly to be preferred to repeated parenteral doses of prophylactic or "on-demand" anti-emetic drugs, or to daily courses of high dose i.v. metoclopramide.

In this trial we placed emphasis on the patients' subjective assessment of their gastro-intestinal symptoms, including retching which is not usually assessed but which some patients find as much if not more distressing than vomiting. In the objective assessment we did not find the measurement of volume of vomitus to be sufficiently reliable to include in the analysis, as others have attempted (Gralla *et al.*, 1981); the volume is also probably of less relevance to the patients than the number of vomiting or retching episodes.

Nabilone was superior in most of the evaluated parameters to oral prochlorperazine, which confirms the findings of two earlier studies. Unlike the subjects of Herman *et al.* (1979), our patients and those of Steele *et al.* (1980) did not significantly prefer nabilone overall to prochlorperazine for subsequent chemotherapy. Since ours was a fixed-dose study, it is not possible to say whether a reduction in dose for patients experiencing troublesome side-effects would have reduced these and improved subsequent acceptability whilst retaining anti-emetic efficacy. Single doses of nabilone in man have been shown to have dose-related pharmacological effects, with 1 mg inducing relaxation and sedation but no dry mouth or hypotension (Lemberger & Rowe, 1975).

The use of widely varying doses of both nabilone and prochlorperazine in published studies has made the interpretation of efficacy and toxicity more difficult. Herman *et al.*, (1979) actually reported on two trials which were analysed together, in one of which patients received 6 mg of nabilone and 30 mg of prochlorperazine daily, and in the other 8 mg of nabilone and 40 mg of prochlorperazine daily. Not surprisingly, the reported toxicity of nabilone was higher than that observed by Steele *et al.* (1980) and our group who used a dose of 4 mg daily, and prochlorperazine at 20 mg and 30 mg daily respectively; the range and incidence of side-effects in these trials are more comparable. The results Steele *et al.* (1980) obtained for nabilone against non-platinum agents and low-dose platinum were also similar to ours. In their pilot study of nabilone against a variety of chemotherapeutic drugs including platinum, Cornbleet *et al.* (1982) used a higher starting dose of 2 mg 6-hourly for the first 12 h, followed by 2 mg 12-hourly. This increase in dose was sufficient for the authors to find a "high incidence (55%) of significant psychotropic side-effects."

We did not find any statistical association between age or sex and nabilone toxicity and so we suggest that the dose of nabilone be restricted to 2 mg 12-hourly for most patients for adequate anti-emetic control aganist non-platinum drugs, with a moderate but overall acceptable incidence of side-effects. Indeed several patients at this dose reported pleasant mental changes, and our limited experience is that subsequent courses of nabilone at the same or lower dose (1 mg 12-hourly) retained its efficacy with no increase in toxicity. However, the long-term use of nabilone has yet to be studied.

Postural hypotension and tachycardia are important pharmacological effects of the cannabinoids (Anonymous, 1978), but this only became a symptomatic problem in 7% of our nabilone subjects. One of the 2 patients with severe cardiovascular toxicity had a mitral valve replacement; extra caution is therefore indicated when using nabilone in patients with known cardiac disease.

Furthermore, unpredictability of the adverse effects discussed above demands the exercise of caution in *all* patients receiving nabilone for the first time, and we would recommend that they receive the drug under in-patient supervision at least for the first 24 hours.

We thank Drs G. Boyd, R.D. Stevenson and S.W. Banham for allowing us to study their patients; Mr J. Urwin of the Pharmacy Department of Glasow Royal Infirmary for practical assistance; Miss Seonaid Buchanan and Miss Irene Adams for secretarial services; and Mrs Rosemary McCusker for the statistical computation. Nabilone and placebo capsules were supplied by Lilly Research Ltd.

### References

ANONYMOUS (1978). Cannabis and the cardiovascular system. *Br. Med. J.*, **i,** 460.

ANONYMOUS (1979). Cancer chemotherapy—the inbuilt deterrent. *Br. Med. J.*, **ii,** 1312.

CORNBLEET, M.A., HAMILTON, D.A., CHRISTIAN, P. & SMITH, J.F. (1982). Evaluation of nabilone as an anti-emetic. *Br. J. Cancer,* **46,** 492.

FRYTAK, S. & MOERTEL, C.G. (1981). Management of nausea and vomiting in the cancer patient. *J. Am. Med. Assoc.*, **245,** 393.

FRYTAK, S., MOERTEL, C.G., O'FALLON, J.R. & 5 others. (1979). Delta-9-tetrahydrocannabinol as an anti-emetic for patients receiving cancer chemotherapy. *Ann. Intern. Med.*, **91,** 825.

GRALLA, R.J., ITRI, L.M., PISKO, S.E & 6 others. (1981). Anti-emetic efficacy of high-dose metoclopramide: randomized trials with placebo and prochlorperazine in patients with chemotherapy-induced nausea and vomitting. *N. Engl. J. Med.*, **305,** 905.

HERMAN, T.S., EINHORN, L.H., JONES, S.E. & 8 others. (1979). Superiority of nabilone over prochlorperazine as an anti-emetic in patients receiving cancer chemotherapy. *N. Engl. J. Med.*, **300,** 1295.

LASZLO, J. (1979). Tetrahydrocannabinol: From pot to prescription? *Ann. Intern. Med.*, **91,** 916.

LASZLO, J. & LUCAS, V.S. (1981). Emesis as a critical problem in chemotherapy. *New Engl. J. Med.*, **305,** 948.

LEMBERGER, L. & ROWE, H. (1975). Clinical pharmacology of nabilone, a cannabinol derivative. *Clin. Pharmacol. Thera.*, **18,** 720.

SALLAN, S.E., ZINBERG, N.E. & FREI, E. (1975). Anti-emetic effect of delta-9-tetrahydrocannabinol in patients receiving cancer chemotherapy. *N. Engl. J. Med.*, **293,** 795.

STEELE, N., GRALLA, R.J., BRAUN, D.W. & YOUNG, C.W. (1980). Double-blind comparison of the anti-emetic effects of nabilone and prochlorperazine on chemotherapy-induced emesis. *Cancer Treat. Rep.*, **68,** 219.

TROUNCE, J.R. (1982). Anti-emetics and cytotoxic drugs. *Br. Med. J.*, **286,** 327.