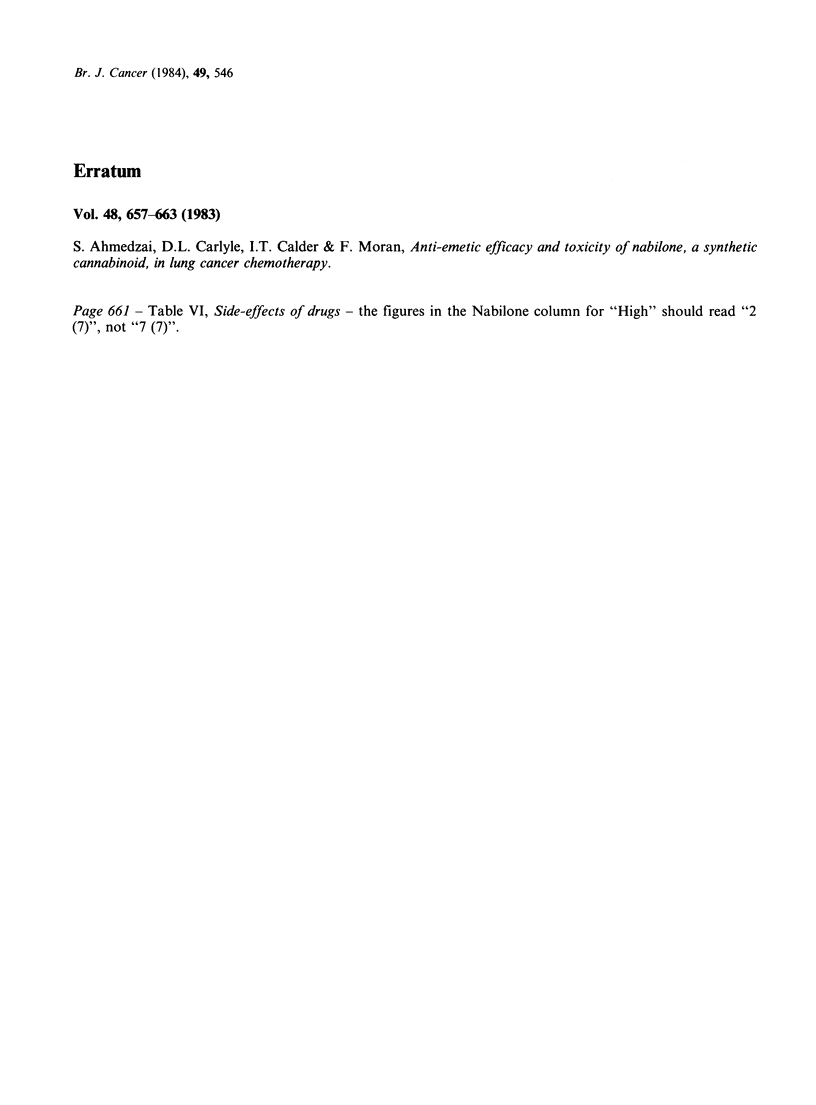# Erratum

**Published:** 1984-04

**Authors:** 


*Br. J. Cancer* (1984), **49**, 546

### Erratum

**Vol. 48, 657–663 (1983)**

S. Ahmedzai, D.L. Carlyle, I.T. Calder & F. Moran, *Anti-emetic efficacy and toxicity of nabilone, a synthetic cannabinoid, in lung cancer chemotherapy.*

*Page 661* – Table VI, *Side-effects of drugs* – the figures in the Nabilone column for "High" should read "2 (7)", not "7 (7)".